# Aquatic Toxicity Comparison of Silver Nanoparticles and Silver Nanowires

**DOI:** 10.1155/2015/893049

**Published:** 2015-06-01

**Authors:** Eun Kyung Sohn, Seyed Ali Johari, Tae Gyu Kim, Jin Kwon Kim, Ellen Kim, Ji Hyun Lee, Young Shin Chung, Il Je Yu

**Affiliations:** ^1^Institute of Nanoproduct Safety Research, Hoseo University, Asan, Republic of Korea; ^2^Fisheries Department, Natural Resources Faculty, University of Kurdistan, P.O. Box 416, Sanandaj 66177-15175, Iran

## Abstract

To better understand the potential ecotoxicological impact of silver nanoparticles (AgNPs) and silver nanowires (AgNWs) released into freshwater environments, the toxicities of these nanomaterials were assessed and compared using Organization for Economic Cooperation and Development (OECD) test guidelines, including a “*Daphnia* sp., acute immobilization test,” “Fish, acute toxicity test,” and “freshwater alga and cyanobacteria, growth inhibition test.” Based on the estimated median lethal/effective concentrations of AgNPs and AgNWs, the susceptibility to the nanomaterials was different among test organisms (daphnia > algae > fish), suggesting that the AgNPs are classified as “category acute 1” for *Daphnia magna*, “category acute 2” for *Oryzias latipes*, and “category acute 1” for *Raphidocelis subcapitata*, while the AgNWs are classified as “category acute 1” for *Daphnia magna*, “category acute 2” for *Oryzias latipes*, and “category acute 2” for *Raphidocelis subcapitata*, according to the GHS (Globally Harmonized System of Classification and Labelling of Chemicals). In conclusion, the present results suggest that more attention should be paid to prevent the accidental or intentional release of silver nanomaterials into freshwater aquatic environments.

## 1. Introduction

The number of engineered nanomaterial (NM) products is increasing in many industrial fields, and large quantities of NM are being released, and eventually entered into the environment. Keller et al. [1] estimated that 63–91% of over 260,000–309,000 metric tons of NMs produced globally in 2010 was ended up in landfills, while the balance was released into soil (8–28%), water bodies (0.4–7%), and atmosphere (0.1–1.5%). Among many different types of NMs, nanoscale silver has been regarded as one of the most important nanomaterials because of its unique properties and ability to form diverse nanostructures, and thus is placed in the list of consumer product inventories [2]. Annually about 63 tons of nanosilver are expected to enter water bodies on a worldwide basis [1] and the concentration in aqueous environments has been predicted to range from 0.03 to 0.32 micrograms per liter [3]. Therefore, understanding the potential harmful effects of nanosilver on aquatic organisms is particularly of importance.

Although silver nanoparticles (AgNPs) and silver nanowires (AgNWs) are basically synthesized from the silver element, their applications in consumer products are different according to their unique properties. The antimicrobial properties of AgNPs are the main reason for their extensive use [4], while the high electrical and thermal conductivities of AgNWs have led to their application in many consumer products such as conductive inks, transparent films, conductive polymer composites, and coatings. In addition, AgNWs possess an effective antimicrobial capability [5].

Although many studies have already investigated the toxic effects of AgNPs in several aquatic organisms such as fish [6],* Daphnia* [7], and algae [8], limited information is currently available on the aquatic toxicity of AgNWs. George et al. [9] demonstrated that Ag nanoplates showed a higher level of toxicity to the rainbow trout gill epithelial cell line and zebrafish embryos when compared to nanospheres and NW in spite of the lower rates of dissolution and bioavailability of this material shape. Also, Kim et al. [10] investigated the gender-specific accumulation of AgNPs in kidneys of Fischer 344 rats, and Artal et al. [11] showed that the acute toxicity of silver vanadate nanowires decorated with AgNPs to* Daphnia similis* was due to the Ag^+^ released from nanomaterials trapped in the gut, along with the Ag released into the test media. Finally, Scanlan et al. [12] recently compared the toxicities of silver ions and nanowires and found that some AgNWs were highly toxic to* D. manga*, yet less toxic than ionic silver. The toxicity of AgNWs varied as a function of their dimensions, coating, and solution chemistry.

Accordingly, the present study investigated the aquatic toxicity of AgNWs (as one-dimensional (1D) silver nanostructure) to three model aquatic organisms, including a fish (*Oryzias latipes*), crustacean (*Daphnia magna*), and algae (*Raphidocelis subcapitata*) according to the Organization for Economic Cooperation and Development (OECD) test guidelines, and compared with the aquatic toxicity of AgNPs (as zero-dimensional (0D) silver nanostructure). The results of study could provide valuable information about potential AgNWs toxicity to aquatic organisms, which might be useful for assessing ecological risk of AgNWs.

## 2. Materials and Methods

### 2.1. Nanomaterials and Characterization

The colloidal AgNPs were purchased from ABC Nanotech Co. Ltd. (Daejeon, Korea). According to information provided by the manufacturer, it contained 20~21 wt% citrate as a capping agent of AgNPs with a diameter range of 5–25 nm. The AgNWs suspension was purchased from Sigma Aldrich (St. Louis, USA), and according to information provided by the manufacturer, it contained 0.5 wt% AgNWs in isopropanol with a diameter of 60 ± 10 nm and length of 10 ± 5 *μ*m. Size distribution of silver nanomaterials (AgNMs) was obtained using dynamic light scattering (DLS) and transmission electron microscope (TEM). DLS provides hydrodynamic size as well as size distribution of nanoparticles in liquid medium. The DLS measurement was conducted with a Nanophox (Sympatec, Clausthal-Zellerfeld, Germany) at 25°C with a complex refraction index of 1.33 and a viscosity of 0.89 mPas. DLS measures the scattering intensity *I*(*q*, *t*) of a sample's Brownian motion, and the autocorrelation function is acquired from *G*_2_(*τ*) = 〈*I*(0)*I*(*τ*)〉 = 1/*T*∫*I*(*t*)*I*(*t* + *τ*)*dt*. When *b* is the experimental constant, the relationship of the normalized first-order autocorrelation function *G*_1_(*τ*) is estimated by *G*_2_(*τ*) = 1 + *b*|*G*_1_(*τ*)|^2^. *G*_1_(*τ*) is then connected to the diffusion constant *D*, *G*_1_(*τ*) = exp(−2*q* ^2^ *Dt*), where *q* = (4*pn*/*l*_0_)sin(*q*/2). In this equation, *n*, *l*_0_, and *q* are the refractive index of the solution, wavelength of the incident light, and scattering angle, respectively. The sample size was estimated using the Einstein-Stokes relation, *D* = *k*_*B*_ *T*/6*phr*, where *k*_*B*_, *T*, and *r* are the Boltzmann factor, temperature, and hydrodynamic radius of the samples [13].

The TEM analysis of the AgNMs was performed using a field emission-transmission electron microscope (FE-TEM, JEM2100F, JEOL, Tokyo, Japan) with an acceleration voltage of 200 kV. Using photographs at a magnification of 100,000, the dimensions of 272 randomly selected AgNPs and 471 randomly selected AgNWs were measured. In addition, the EDX analysis of the AgNMs was performed using an energy-dispersive X-ray spectrometer (EDS, TM200, Oxford, UK). TEM samples were prepared as diluted using DI water.

### 2.2. Silver Concentration in Test Suspension

The actual concentrations of silver in the fish toxicity suspension were analyzed using an atomic absorption spectrophotometer equipped with a Zeeman graphite furnace (Perkin Elmer 5100ZL, Zeeman Furnace Module, USA) based on the NIOSH 7300 method [14].

### 2.3. Fish Toxicity Tests

Three-month* Oryzias latipes* juveniles with a mean total body weight of 0.39 ± 0.01 g (mean ± SD) and mean total body length of 2.62 ± 0.05 cm (mean ± SD) were used for the toxicity experiments. Prior to the experiments, fish were kept in 100 L tanks with a water circulation system and 16/8 hour light and dark cycle and were fed pellet feed (TOPMEAL) at 1% of their body weight. After 7 days of adaptation, fish were transferred to separate test vessels with a volume of 10 L and allowed to adapt for a further 24 hrs prior to starting the toxicity experiments. Dechlorinated tap water was used for all experiments (dechlorination performed by vigorous aeration for at least 48 hrs). To avoid overestimating the toxicity, the feeding of the fish was stopped 48 hrs before starting the experiments in order to minimize the risk of AgNMs absorption in the fecal material or food and minimize the dissolved organic carbon (DOC) in the exposure tanks [15].

The acute (96 hrs) toxicity tests were conducted on fish in accordance with standard OECD guideline number 203 (Fish, acute toxicity test) [16]. A series of preliminary experiments (10, 1, 0.1, and 0.01 mg/L) was conducted to determine the range of AgNMs concentrations that produced mortality of the fish. As a result, nominal concentrations of 10, 5, 2.5, 1.25, 0.625, and 0.312 mg/L were selected as effective concentrations for performing the main toxicity tests of each chemical. The fish were exposed to the AgNMs based on a static exposure regime. For every experiment, 7 healthy fishes were directly transferred into each prepared concentration. Control groups (7 fishes) were also included for each treatment. An additional vehicle control group containing isopropyl alcohol (IPA, 0.2%) was included along with the AgNWs experiments. The mortalities were recorded at 24, 48, 72, and 96 hours postexposure and the LC_50_ values were calculated using a probit analysis (PASW statistics 18, 2009, SPSS INC, Chicago, IL).

### 2.4. *Daphnia* Toxicity Tests

The acute (48 h) toxicity tests were conducted on neonate* Daphnia magna* (younger than 24 hrs old) in accordance with standard OECD guideline number 202 (*Daphnia* Sp. acute immobilization test) [17]. Here, fully aerated M4 media were used as the exposure media and the test solutions were prepared immediately before use by diluting appropriate amounts of the AgNMs in the M4 media. All tests were conducted in a water bath system with a constant temperature (21.45 ± 0.10°C) and 16/8 hrs light dark cycle. In the experiments, dissolved oxygen, pH, hardness, and alkalinity of exposure media (mean ± SD) were 4.87 ± 0.14 mg/L, 7.47 ± 0.01, 140 mg/L, and 230 mg/L, respectively. Since the presence of algae was shown to affect the toxicity of AgNPs [18] and presence of organic matter found to inhibit the Ag ion uptake by* Daphnia* [19], animals were not fed during the experiments.

A series of preliminary experiments (1, 0.1, 0.01, and 0.001 mg/L) was conducted to determine the concentration ranges of each AgNM that produced immobility of* D. magna*. According to the determined concentration ranges, effective concentrations were then selected (0.0161, 0.0115, 0.0082, 0.0059. 0.0042, and 0.0030 mg/L for AgNPs; 0.32, 0.16, 0.08, 0.04, 0.02, 0.01, and 0.005 mg/L for AgNWs). The neonates were exposed to each AgNM in four repeats (5 neonates per replicate) based on a static exposure regime in 100 mL of the exposure media in glass exposure beakers. Control groups (5 neonates in four repeated) were also included for each treatment. An additional vehicle control group containing isopropyl alcohol (IPA, 0.24%) was included in the AgNWs experiments. After 24 and 48 hrs of exposure, immobilization and mortality of the* Daphnia* were assessed in each test beaker. According to Annex 1 of OECD guideline number 202, an animal was recorded as dead when it was immobile, that is, not able to swim or no observed movement of appendages or the postabdomen within 15 sec after agitation of the test container [17]. Furthermore, the live* Daphnia* were categorized according to their swimming type: normal swimming (NOR), abnormal swimming (ABN), erratic swimming (ERR),* Daphnia* mainly at the bottom (BOT), and* Daphnia* mainly at the surface (SUR) [7]. Any visible uptake or adsorption of NM by the* D. magna* was also monitored and recorded. The EC_10_, EC_50_, and EC_90_ values were calculated using a probit analysis (PASW statistics 18, 2009, SPSS Inc, Chicago, IL).

### 2.5. Algae Toxicity Tests

The acute (72 hrs) toxicity tests of the AgNMs were conducted on* Raphidocelis subcapitata*, ATCC 22662 (formerly known as* Pseudokirchneriella subcapitata* and* Selenastrum capricornutum*) in accordance with standard OECD guideline number 201 (freshwater alga and cyanobacteria, growth inhibition test) [20].

Algal cells in the exponential growth phase were used for all the experiments. At the start of each experiment 10,000 cells/mL were added to 100 mL of each test medium under sterile conditions. The pH of the prepared culture media was first adjusted to a final value of 7.32 ± 0.06 using 0.1 N NaOH or 0.1 N HCl solutions. Each growth-inhibition test consisted of 6 AgNPs and AgNWs test concentrations (8, 4, 2, 1, 0.5, and 0.25 mg/L) with triplicate for each concentration. Triplicate control groups (0 mg/L AgNPs and AgNWs) and triplicate vehicle control groups (0.24% IPA) were used for comparison. The test vessels were incubated for 72 hrs under continuous illumination (5.44 ± 0.53 lux) at a constant temperature of 24°C in an automatic shaking incubator with illumination. After 24, 48, and 72 h of exposure, the cell density (biomass) for each treatment was determined using a particle counter, while the values for the logarithmic growth rate (*Y*), biomass at time *t* (*Xt*), percent inhibition of yield (Iy), mean value for yield in the control group (Yc), value for yield for the treatment replicate (Yt), average specific growth rate from time 0 to 72 hr (*μ*_0−72_), mean value for the average specific growth rate in the control group (*μ*_*c*_), average specific growth rate for the treatment replicate (*μ*_*t*_), and percent inhibition of the average specific growth rate (Ir) were all obtained using the following formulas:

(1) $$\begin{array}{c} Y=X_{72}-X_{0} \end{array}$$

(2) $$\begin{array}{c} \%Iy=\left\{\frac{\left(Yc-Yt\right)}{Yc}\right\}\times 100 \end{array}$$

(3) $$\begin{array}{c} \mu_{0-72}=\frac{\left(lnX_{72}-lnX_{0}\right)}{72} \end{array}$$

(4) $$\begin{array}{c} \%Ir=\left\{\frac{\left(\mu_{c}-\mu_{t}\right)}{\mu_{c}}\right\}\times 100. \end{array}$$

The EC_50_ values were calculated using a probit analysis (PASW statistics 18, 2009, SPSS Inc., Chicago, IL). In addition, the morphology of the algal cells was observed following each treatment and categorized as normal (NOR), swollen (SWO), flocculated (FLC), decolored (DEC), ruptured (RUP), and/or atrophied (ATR).

### 2.6. Statistical Analysis

Data of all experiment were analyzed using the PASW statistic (Ver. 18, 2009, SPSS Inc., Chicago, IL, USA) and presented as mean ± standard deviation. The LC_50_ values of fish test during 96 hrs, EC_50_ values of daphnia 48 hrs and alga 72 hrs were calculated by a probit analysis method. Comparison of nominal and actual concentrations of silver in fish toxicity test after 3 hrs versus 96 hrs was performed using Student's *t*-test. Difference were considered statistically significant when ^*∗*^ *P* < 0.05.

## 3. Results

### 3.1. Particle Characterization

**Figure 1:**
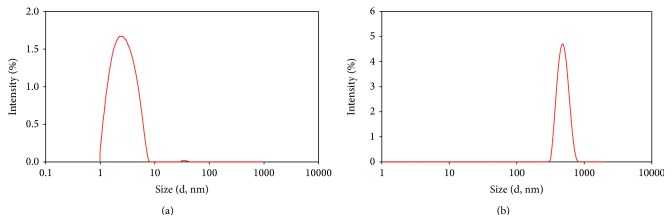
Size distribution of the AgNPs ((a) 5 mg/L) and AgNWs ((b) 100 mg/L) by DLS (d hydrodynamic diameter).

The AgNPs and AgNWs size and distribution were measured by DLS before aquatic toxicity test.  Figure 1 shows the hydrodynamic size distribution of colloidal AgNPs and AgNWs diluted in DI water.  Figure 1(a) shows the hydrodynamic size distribution of AgNPs by intensity, and the size of AgNPs was distributed up to 10 nm with mean hydrodynamic diameter 2.36 nm.  Figure 1(b) shows hydrodynamic size distribution of AgNWs with mean diameter 2.1 *μ*m.

**Figure 2:**
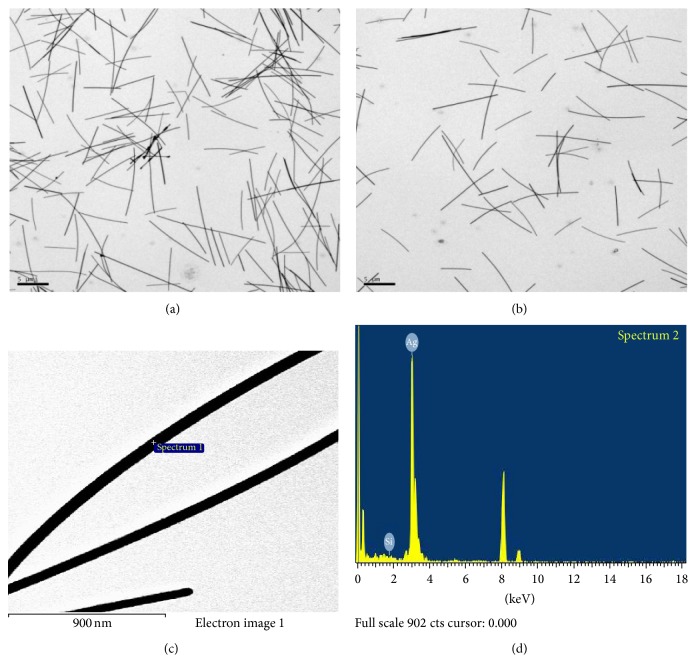
AgNWs, (a)-(b) TEM morphology and (c)-(d) EDX spectrometer pattern.

In the case of the AgNWs diluted in fish test media (dechlorinated tap water) observed by TEM (Figures 2(a) and 2(b)), the count median length (CML) and geometric standard deviation (GSD) of the wires were 7.4 *μ*m and 1.5, respectively (Figure 4(a)). The average diameter and standard deviation (SD) of the AgNW in TEM images were 57.01 ± 1.2 nm. Since the AgNPs and AgNWs preparations were in liquid, it was not possible to measure Brunauer Emmett and Teller (BET) surface area. Thus the average surface area measured from TEM image of the AgNWs was 6.37 × 10^18^ nm^2^/g.

**Figure 3:**
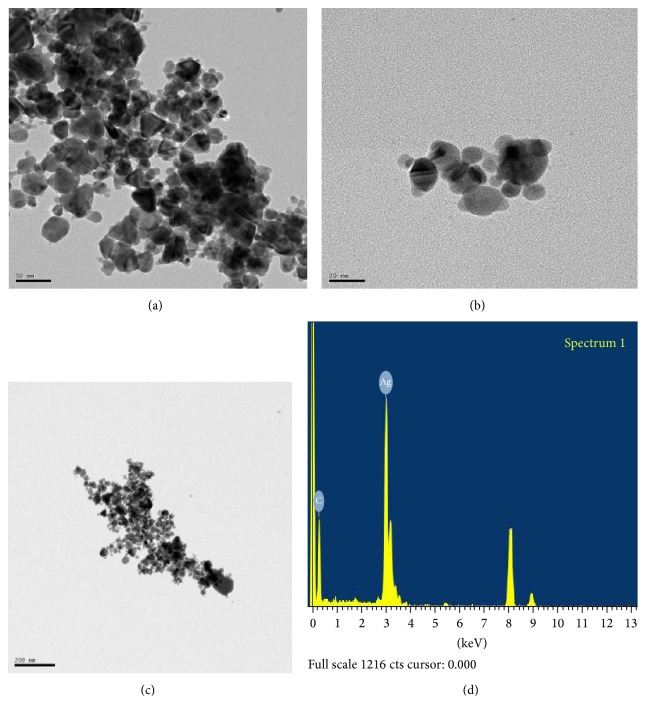
AgNPs, (a)-(b) TEM morphology and (c)-(d) EDX spectrometer pattern. The scale bar in (a) indicates 50 nm, (b) for 20 nm and (c) for 200 nm.

**Figure 4:**
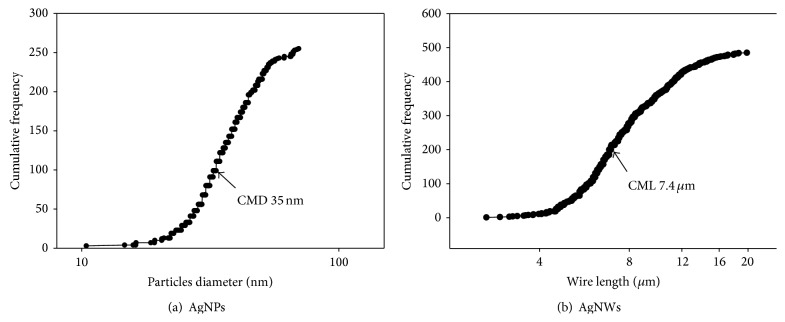
Size distribution of wires/particles based on cumulative frequency determined from transmission electron microscope data for (a) AgNPs and (b) AgNWs.

In the case of the AgNPs diluted in fish test media observed by TEM, the particles were spherical in shape (Figures 3(a) and 3(b)), with a count median diameter (CMD) of 35 nm (Figure 4(b)). Plus, the GSD of the colloidal AgNPs was 1.4. The average measured surface area of the AgNPs was 1.63 × 10^19^ nm^2^/g (more 2-fold greater than the average surface area of the AgNWs).

As seen in Figures 2(c), 2(d), 3(c) and 3(d), the energy dispersive X-ray (EDX) analyses revealed the presence of elemental silver in both AgNWs and AgNPs colloids.

### 3.2. Silver Concentrations in Test Suspensions for Fish Toxicity Testing

The concentrations of AgNPs and AgNWs in the test suspensions were determined after 3- and 96-hour test period. After 3 hrs, the concentrations of silver in the nanoparticle and nanowire suspensions were well maintained with only a slight deviation from the nominal concentrations (Table 1(a)). However, after 96 hrs, the silver concentrations showed a higher deviation from the nominal concentrations and silver concentrations after 3 hrs (Table 1(b)). This greater deviation was due to agglomeration/aggregation or precipitation of the AgNPs and AgNWs.

### 3.3. Toxicity of AgNMs in Fish

**Figure 5:**
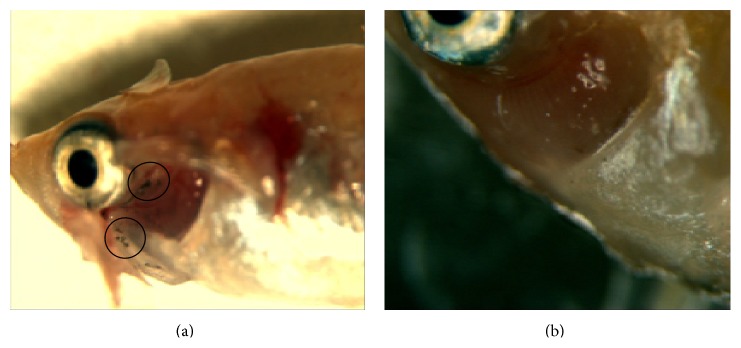
(a) Signs of AgNPs accumulation on fish gills during 96 hr. (b) No visible accumulation of AgNWs. The circles indicate accumulation of AgNPs on the fish gill.

During the experiments, the mean and SD of the water pH, temperature, and dissolved oxygen in the exposure tanks were 7.85 ± 0.13, 21.7 ± 0.61°C, and 8.8 ± 0.17 mg/L, respectively. No mortality was observed either in the control or the vehicle control during the experimental period. The average 96-hour median lethal concentrations (LC_50_) of AgNPs and AgNWs for* Oryzias latipes* were estimated to be 1.8 and 4.18 mg/L, respectively. From these results, the LC_50_ proportion of AgNWs to AgNPs was 2.32, indicating AgNPs were estimated to be 2.32 times more toxic than the AgNWs for* Oryzias latipes*. Although signs of AgNPs accumulation were visible in fish gills, no apparent accumulation was detected after AgNWs exposure (Figure 5).

### 3.4. Toxicity of AgNMs in* Daphnia*

During the exposure period, the control groups showed zero mortality for all the experiments. The average values of the effective concentrations are shown in Table 2. The median effective concentrations (EC_50_s) of AgNPs and AgNWs were calculated as 0.012 and 0.139 mg/L, respectively. From these results, the EC_50_ proportion of AgNWs to AgNPs was 11.58, meaning the AgNPs were estimated to be 11.58 times more toxic than the AgNWs for* Daphnia magna*.

The normal and abnormal swimming of the live* Daphnia* are summarized in Table 3. For all the control and vehicle control groups, 100% of the live* Daphnia* exhibited completely normal swimming. For the AgNWs treatments, at concentrations up to 0.080 mg/L, 100% of the live* Daphnia* exhibited normal swimming, while at 0.16 and 0.32 mg/L, 90% and 100% of the* Daphnia* exhibited abnormal swimming, respectively. All the abnormalities in the AgNWs groups were related to erratic swimming (ERR). Meanwhile, for the AgNPs treatments, at concentrations of 0.0059 and lower, all the* Daphnia* exhibited normal swimming, whereas, at higher concentrations (0.0082 and 0.0115 mg/L), the* Daphnia* started to exhibit abnormal swimming, and, at 0.0161 mg/L, all the* Daphnia* exhibited abnormal swimming. Most of the abnormalities in the AgNPs groups were related to erratic swimming (ERR), although 10% of the abnormal* Daphnia* at 0.0115 mg/L were mainly at the bottom (BOT). No* Daphnia* mainly at the surface (SUR) were observed in this study.

**Figure 6:**
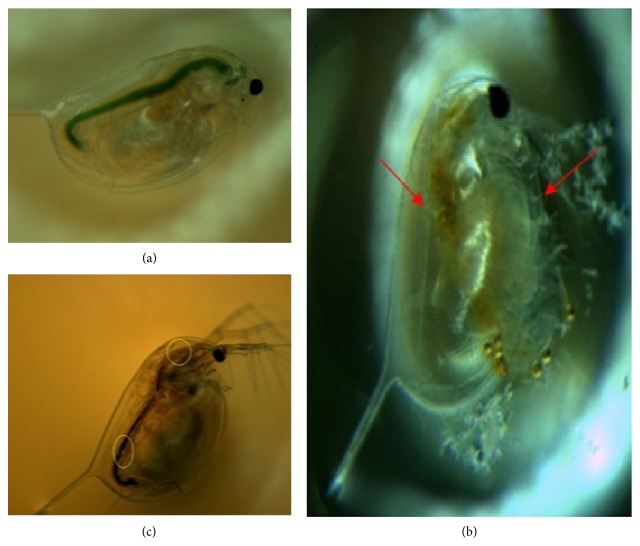
Microscope images of (a) control* Daphnia* fed with chlorella, (b)* Daphnia* exposed to AgNPs, and (c)* Daphnia* exposed to AgNWs. The arrows of (b) indicate accumulation of AgNP in the gut (left) and on the antenna (right) of* Daphnia magna*. The circles of (c) indicate accumulation of AgNWs in the gut of* Daphnia magna*.

A notable phenomenon with the AgNPs treatments was the appearance of small bubbles under the carapace of the* Daphnia* (Figure 6(b)). This pigmentation became visible in parts of the brood chamber that was not observed in the controls (Figure 6(b)); this pigmentation may have been a sign of nanoparticle accumulation under the carapace. As regards the AgNWs treatments, large amounts of ingested nanowires were found in the gut tract of the* Daphnia* (Figure 6(c)).

### 3.5. Toxicity of AgNMs in Alga

**Figure 7:**
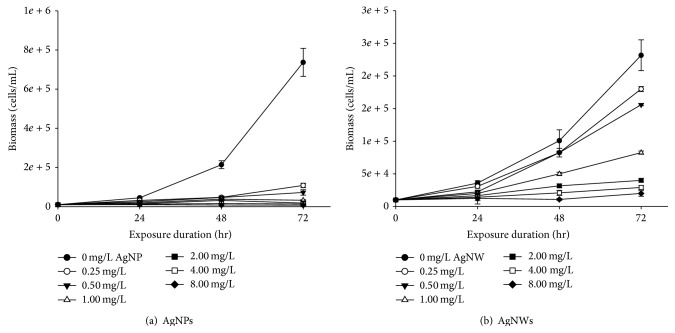
Growth inhibition of biomass caused by AgNWs and AgNPs. Error bars indicate standard deviation.

**Figure 8:**
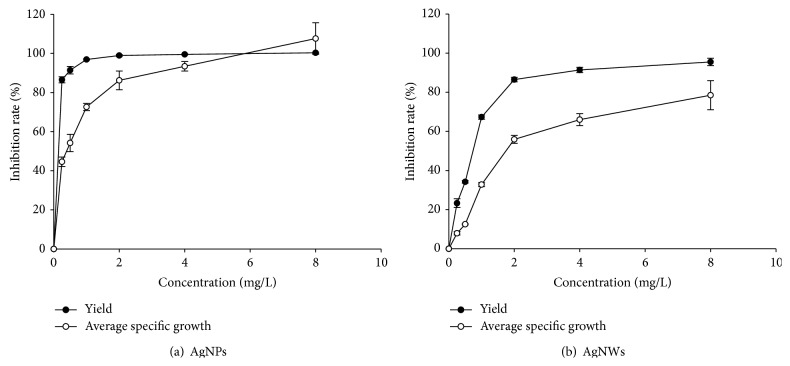
Inhibition rate of average specific growth and yield. Error bars indicate standard deviation.

The growth inhibition of the cell biomass of* Raphidocelis subcapitata* during 72 hrs of exposure to different concentrations of AgNWs and AgNPs are shown in Figure 7. The cell biomass increase rate, logarithmic growth rate, average specific growth rate, percent of yield inhibition, and percent of average specific growth rate inhibition are all summarized in Table 4 and Figure 8, satisfying the validity of the test prescribed in the OECD Test guideline 201 [20]. The OECD 201 states that the biomass in the control cultures should be increased more than 16-fold within the 72-hour test period, and the mean coefficient of variation for section-by-section specific growth rates in the control culture should not exceed 35% in alga test. Also, the coefficient of variation of average specific growth rates during the whole test period in replicate control culture should not exceed 7% in alga tests. The inhibitory rates increased when increasing the concentrations of AgNMs. While all the cells in the control groups retained a normal appearance, AgNWs and AgNPs concentration of 0.25 mg/L resulted in flocculation and 0.5 mg/L and higher concentrations resulted in flocculation, depolarization, rupture, and atrophy. None of the AgNWs and AgNPs concentrations produced any swelling.

The average values for the median effective concentrations (EC_50_), no observed effect concentrations (NOEC), and lowest observed effect concentrations (LOEC) are shown in Table 5. The 72 hrs EC_50_ (concentration at which a 50% inhibition of the growth rate is observed) of AgNWs and AgNPs for the average specific growth rate of* Raphidocelis subcapitata* was calculated as 2.573 mg/L and 0.74 mg/L, respectively.

## 4. Discussion

The AgNMs were characterized using DLS and TEM. The measured AgNPs by DLS obtained that result is similar to information provided by the manufacturer (Figure 1(a)). In case of AgNWs, that result is slightly different from the information provided by the manufacturer (Figure 1(b)). Hydrodynamic diameter measured by DLS could be appropriate for spherical with monodispersed particles [21]. DLS has been used to measure particle size distribution of multiwall carbon nanotubes [22], although the size distribution is not exactly comparable to TEM size distribution. In this study TEM and DLS size are compared. As expected, particle size by DLS, which measures hydrodynamic size, is larger than TEM size.

This study demonstrated that AgNPs and AgNWs are both toxic to fresh water organisms, including fish (*Oryzias latipes*), water fleas (*Daphnia magna*), and algae (*Raphidocelis subcapitata*). Based on the present results, the calculated LC_50_ or EC_50_ of AgNPs for the fish, daphnia, and algae were about 2.3-, 11.5-, and 3.5-fold lower, respectively, than the corresponding values of AgNWs. Thus, AgNPs would seem to be more toxic to aquatic organisms than AgNWs, at least in freshwater media. Recent studies shown that the dissolved Ag^+^ released from AgNPs and AgNWs plays a critical role in the aquatic toxicity of these nanomaterials [11, 23, 24]. Meanwhile, the degree of Ag^+^ dissolution depends on the surface area of the nanoscale silver, and the present study showed that the average measured surface area of the AgNPs was more than 2-fold greater than that of the AgNWs. The amount of silver ions released from the surface of the AgNPs was greater than the amount of Ag^+^ released from the surface of the AgNWs; thus the AgNPs logically displayed a greater toxicity to the aquatic organisms when compared with the AgNWs. In another recent study, Visnapuu et al. [25] found that the toxicity and bioavailability of AgNWs and AgNPs to* E. coli* depended on the dissolved silver ions with no shape-induced/related effects. Beer et al. [26] showed that free silver ions in AgNPs suspensions play a significant role in the toxicity of AgNPs suspensions to A549 lung cells. Overall, these results with our current observation indicate that silver ions released from particle or wire are toxicity determinants and surface area is major factor in releasing silver ions from particles and wires.

In contrast to the present results, Stoehr et al. [27] showed that silver wires (length: 1.5–25 *μ*m; diameter 100–160 nm) had a strong effect on A549 human lung epithelial cells, whereas PVP-coated spherical silver nanoparticles (30 nm) had no effect with a similar particle mass, surface area, and number concentration. In this case, the PVP coating may have reduced the release of Ag^+^ from the AgNPs, thereby decreasing the toxicity at the tested concentrations, or the shape may have affected the toxicity, as observed with other high aspect ratio nanoparticles (HARN) [28, 29]. When the freshwater mussel* Elliptio complanata* was exposed to increasing concentrations of 20-nm AgNPs, 80-nm AgNPS, and dissolved Ag ions for 48 h at 15°C, the response pattern of 80 nm AgNPS was more closely related to Ag ions than 20 nm AgNPs, suggesting a more important release of dissolved Ag ions from 80 nm AgNPs [30]. These results indicate that some more complicated toxic mechanisms for AgNPs are presented other than silver ion release from the surface of AgNPs or AgNWs.

The same abnormal swimming, brood chamber pigmentation, NM ingestion, and small bubbles under the carapace of the* D. magna* exposed to nanoscale silver observed in the current study were also previously reported by Asghari et al. [7] in the case of* D. magna* exposed to AgNPs. Further, Artal et al. [11] described an increase in the droplet size in* Daphnia* as a response to nanowire exposure that was similar to the bubbles observed in the present study. The large quantities of ingested AgNWs found in the gut tract of the* Daphnia* in the current study were similar to previous reports of the ingestion of other one-dimensional nanostructures that became trapped in the digestive tract [11, 31, 32]. As* Daphnia* are a major food source for many kinds of fish, aquatic insect larvae, and other invertebrates, their ingestion of nanomaterials represents a potential for subsequent transfer of nanomaterials to higher organisms via food chain.

In this paper, comparison is done on aquatic organism toxicity of AgNPs with AgNWs based on the OECD test guidelines 201, 202, and 203. In 2013, OECD Working Party on Manufactured Nanomaterials (WPMN) recommended their member countries to use OECD test guidelines in safety testing for nanomaterials after 6 years of OECD work on the safety of manufactured nanomaterials. Safety of nanomaterials can be addressed with existing test methods and assessment approaches, although some cases might be necessary to adapt methods of sample preparation and dosimetry of safety testing but not necessary to develop completely new approaches for nanomaterials [33]. Thus the test results for AgNPs and AgNWs based on the OECD test guidelines might be very useful in evaluating safety of nanomaterials. Current hazard classification used in the Globally Harmonized System of Classification and Labelling of Chemicals [34] is also based on the test results established on the OECD test guidelines.

According to the median lethal/effective concentrations of AgNWs and AgNPs estimated in the present study, the order of animal susceptibility was daphnia > algae > fish. Based on the GHS hazard classification, the AgNPs and AgNWs tested in this study should both be classified as “category acute 1” for* D. magna* and “category acute 2” for* Oryzias latipes*. Plus, the AgNWs and AgNPs should be classified as “category acute 2” and “category acute 1” for* Raphidocelis subcapitata,* respectively. Consequently, based on the current findings and the results of other published studies, AgNMs would appear to have a toxic effect on aquatic organisms, at least in freshwater environments; thus more attention should be paid to preventing their accidental or intentional release into aquatic ecosystems.

## Figures and Tables

**Table:** (a) AgNWs

Nominal concentration (mg/L)	0	0.313	0.625	1.250	2.5	5	10
Actual concentration after 3 hr	0	0.37 ± 0.05	0.71 ± 0.03	1.10 ± 0.04	3.68 ± 0.53	7.34 ± 0.68	12.39 ± 1.10
Actual concentration after 96 hr	0	0.31 ± 0.04	0.55 ± 0.06^**^	0.93 ± 0.06^**^	3.06 ± 0.24	5.35 ± 0.56^**^	11.34 ± 0.63

^*∗∗*^ *P* < 0.01, 3 hr versus 96 hr by Student's t-test. Data are presented as mean ± standard deviation.

**Table:** (b) AgNPs

Nominal concentration (mg/L)	0	0.313	0.625	1.250	2.5	5	10
Actual concentration after 3 hr (n = 3)	0	0.32 ± 0.02	0.53 ± 0.04	1.03 ± 0.06	2.27 ± 0.31	4.89 ± 0.43	10.29 ± 0.51
Actual concentration after 96 hr (n = 3)	0	0.24 ± 0.02^**^	0.66 ± 0.05^*^	0.82 ± 0.07^*^	1.99 ± 0.25	4.13 ± 0.48	8.26 ± 0.78^*^

^*∗*^ *P* < 0.05, 3 hr versus 96 hr; ^*∗∗*^ *P* < 0.01, 3 hr versus 96 hr by Student's t-test. Data are presented as mean ± standard deviation.

**Table 2:** Effective concentration (EC) values of AgNPs and AgNWs for *Daphnia magna* neonates during 48 hrs.

Nanomaterial	Average EC_10_ (mg/L)	Average EC_50_ (mg/L)	Average EC_90_ (mg/L)
AgNWs	0.117	0.139	0.160
AgNPs	0.007	0.012	0.017

**Table 3:** Percentage of normal (NOR) and abnormal (ABN) *Daphnia magna* neonates during 48-hour exposure to AgNPs and AgNWs. ERR: erratic swimming, BOT: *Daphnia* mainly at the bottom, and SUR: *Daphnia* mainly at the surface.

Nanomaterial	Conc. (mg/L)	%NOR	%ABN	%ERR	%BOT	%SUR

AgNWs	(Control) 0	100	0	0	0	0
	Vehicle control	100	0	0	0	0
	0.005	100	0	0	0	0
	0.010	100	0	0	0	0
	0.020	100	0	0	0	0
	0.040	100	0	0	0	0
	0.080	100	0	0	0	0
	0.160	10	90	90	0	0
	0.320	0	100	100	0	0

AgNPs	(Control) 0	100	0	0	0	0
	0.0030	100	0	0	0	0
	0.0042	100	0	0	0	0
	0.0059	100	0	0	0	0
	0.0082	25	75	75	0	0
	0.0115	30	70	60	10	0
	0.0161	0	100	100	0	0

**Table 4:** Rate of biomass increase, average specific growth rate, and yield inhibition of algal cells during 72-hour exposure to different concentrations of AgNWs and AgNPs.

NMs	Conc. (mg/L)	Rate of cell biomass increase	*Y* ^a^	%Iy^b^	*µ*_0–72^c^_	%Ir^d^
AgNWs	Control (0)	22.25 ± 2.47	221,660	0	0.043 ± 0.001	0
	Vehicle control	18.6 ± 1.50	175,830	20.7	0.040 ± 0.001	6.98
	0.25	18 ± 0.5	170,000	23.3	0.040 ± 0.000	7.93
	0.5	15.56 ± 0.11	145,833	34.2	0.038 ± 0.000	12.5
	1	8.23 ± 0.25	72,500	67.3	0.029 ± 0.000	32.8
	2	3.96 ± 0.25	30,000	86.5	0.019 ± 0.000	55.9
	4	3.03 ± 0.28	19,167	91.4	0.014 ± 0.001	66
	8	1.5 ± 1.21	5,500	97.5	−0.001 ± 0.021	103

AgNPs	Control (0)	73.67 ± 7.17	726,667	0	0.060 ± 0.001	0
	0.25	10.83 ± 1.13	98,333	86.5	0.033 ± 0.001	44.6
	0.5	7.25 ± 1.39	62,500	91.4	0.027 ± 0.003	54.2
	1	3.25 ± 0.25	22,500	96.9	0.016 ± 0.001	72.6
	2	1.83 ± 0.38	8,333	98.9	0.008 ± 0.003	86.2
	4	1.33 ± 0.14	3,333	99.5	0.004 ± 0.001	93.4
	8	0.75 ± 0.25	−2,500	100.3	−0.005 ± 0.004	107.6

*Y* ^a^: logarithmic growth rate; %Iy^b^: percent of yield inhibition; *µ*_0–72^c^_: average specific growth rate from time 0 to 72; %Ir^d^: percent of average specific growth rate inhibition. Data are presented as mean ± standard deviation.

**Table 5:** Values of median effective concentration (EC_50_), no observed effect concentration (NOEC), and lowest observed effect concentration (LOEC) of AgNWs and AgNPs for* Raphidocelis subcapitata* during 72 hrs.

NMs	Yield				Average specific growth rate			
	EC_50_ (mg/L)	95% confidence limits (mg/L)	NOEC (mg/L)	LOEC (mg/L)	EC_50_ (mg/L)	95% confidence limits (mg/L)	NOEC (mg/L)	LOEC (mg/L)
AgNWs	1.22	—	—	0.25	2.57	1.55~6.05	0.25	1.00
AgNPs	0.14			0.25	0.74			0.25
